# Insights into monkeypox pathophysiology, global prevalence, clinical manifestation and treatments

**DOI:** 10.3389/fimmu.2023.1132250

**Published:** 2023-03-21

**Authors:** Liyan Niu, Dingfa Liang, Qin Ling, Jing Zhang, Ziwen Li, Deju Zhang, Panpan Xia, Zicheng Zhu, Jitao Lin, Ao Shi, Jianyong Ma, Peng Yu, Xiao Liu

**Affiliations:** ^1^ Department of Endocrinology and Metabolism, The Second Affiliated Hospital of Nanchang University, Nanchang, Jiangxi, China; ^2^ Huan Kui College of Nanchang University, Nanchang, China; ^3^ Queen Mary College of Nanchang University, Nanchang, China; ^4^ Department of Anesthesiology, The Second Affiliated Hospital of Nanchang University, Nanchang, Jiangxi, China; ^5^ Third Department of Internal Medicine, Dexing Hospital of Traditional Chinese Medicine, Dexing, Jiangxi, China; ^6^ Department of Pharmacology and Systems Physiology, University of Cincinnati College of Medicine, Cincinnati, OH, United States; ^7^ School of Medicine, St. George University of London, London, United Kingdom; ^8^ Department of Cardiology, Sun Yat-Sen Memorial Hospital of Sun Yat-Sen University, Guangzhou, Guangdong, China

**Keywords:** monkeypox virus (MPV), origin, pathophysiology, global prevalence, clinical manifestation, treatment

## Abstract

On 23rd July 2022, the World Health Organization (WHO) recognized the ongoing monkeypox outbreak as a public medical crisis. Monkeypox virus (MPV), the etiological agent of monkeypox, is a zoonotic, linear, double-stranded DNA virus. In 1970, the Democratic Republic of the Congo reported the first case of MPV infection. Human-to-human transmission can happen through sexual contact, inhaled droplets, or skin-to-skin contact. Once inoculated, the viruses multiply rapidly and spread into the bloodstream to cause viremia, which then affect multiple organs, including the skin, gastrointestinal tract, genitals, lungs, and liver. By September 9, 2022, more than 57,000 cases had been reported in 103 locations, especially in Europe and the United States. Infected patients are characterized by physical symptoms such as red rash, fatigue, backache, muscle aches, headache, and fever. A variety of medical strategies are available for orthopoxviruses, including monkeypox. Monkeypox prevention following the smallpox vaccine has shown up to 85% efficacy, and several antiviral drugs, such as Cidofovir and Brincidofovir, may slow the viral spread. In this article, we review the origin, pathophysiology, global epidemiology, clinical manifestation, and possible treatments of MPV to prevent the propagation of the virus and provide cues to generate specific drugs.

## 1 Introduction

Threats from the pandemic coronavirus disease 2019 (COVID-19) have not been eliminated, and worries about the possibility of another viral pandemic reached a crescendo (1). The monkeypox virus (MPV), a zoonotic DNA orthopoxvirus (OPV) causes the zoonotic disease monkeypox marked by fever and a red rash which is related to smallpox virus and vaccinia virus (VACV) (2, 3). In 1970, the Democratic Republic of Congo (DRC) reported the first human case of infection, accounting for the majority of early cases (4). Recently, more than 57,000 total cases have been found at more than 100 total locations since May 2022 (5). According to the Centers for Disease Control (CDC), there are no specific medications available, and smallpox vaccines, vaccinia immune globulin, and antivirals were used to regulate small-scale outbreaks (6, 7). Such unexpected, unprecedented, and unusually high-frequency transmission outbreaks worldwide have spurred scientific, political, and media attention. Investigation of pathophysiological features, routes of transmission, clinical features and preliminary diagnosis is urgently needed and has the potential to help improve prevention and early intervention and promote the development of specific drugs.

## 2 Origins and transmission routes

**Figure 1:**
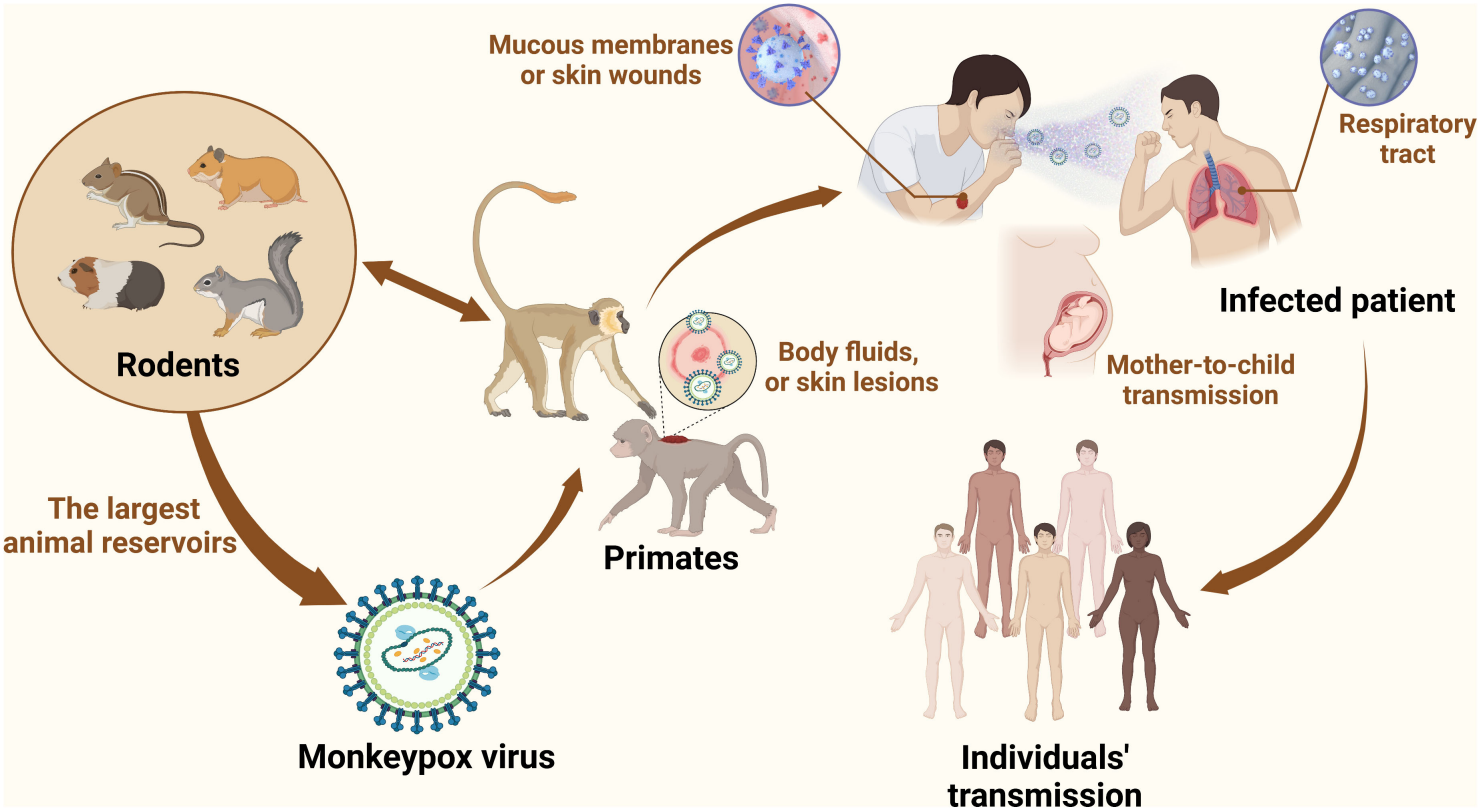
MPV transmission. Zoonotic dissemination of MPV. Animal hosts mainly include rodents (African rope squirrels, prairie dogs, hamsters, and rats) and primates (cynomolgus, rhesus, and gorillas). MPV is an enveloped dsDNA virus belonging to the Poxviridae family, and smallpox is also one of the most prevalent viruses in this family. Wildlife trafficking, habitat degradation, and climate change contribute to the transmission of MPV from new species to other species and increase the bond between people and animals. In the case of wildlife trafficking and illegal hunting, animals are caught, trapped, transported, and sold as food, medicine, and pets. Wildlife trade markets promote disease dissemination, making it possible for viruses from many neighboring species to jump the species barrier. Animal-to-human transmission is mediated by bites, scratches, and slaughtering. Human-to-human transmission occurs through close contact with infected people, such as through respiratory droplets and skin contact, especially MSM. The virus can remain on fomites, such as bedding, linen, and clothes.

In 1958, MPV was first identified and isolated from cynomolgus (*Macaca cynomolgus*) monkeys as laboratory animals, which were kept by a research facility after shipment from Singapore to Denmark (8). MPV is a member of the OPV of the Chordopoxvirinae subfamily, Poxviridae family, the same genus as other viruses such as smallpox and cowpox (9). There are some similarities between monkeypox and smallpox, such as structures, clinical manifestations, and responses to some antiviral drugs (10, 11). The MPV of human cases originated in the tropical rainforests of western and central Africa, and there are two clades in the phylogenetic analysis: the West African clade and the Congo clade, with an average mortality rate of up to 11% (12). The first isolates from cynomolgus (*Macaca cynomolgus*) monkeys belonged to the West African clade while the Congo clade contributed to the first human case in 1970 (13). In addition, monkeypox is a zoonotic disease similar to smallpox, but it is still unclear which is the reservoir host of MPV (14). Rodents from Africa are thought to be the largest animal reservoirs involved in the spread of the virus, and it can be transmitted from some rodents (prairie dogs) to various monkeys and apes, such as anthropoid apes. By coming into contact with the infected animals’ respiratory droplets, skin sores, or body fluids, MPV can transmit from one animal to another. The virus enters a healthy person *via* the respiratory system, mucous membranes (such as the nose, mouth, or eyes), or skin wounds (15). Simultaneously, it can be transmitted from animals, such as rodents or monkeys, to humans through bushmeat, bites, scratches, and direct or indirect exposure to body substances or fluids from the lesion (16) (**Figure 1**). In 1970, the first reported case of MPV was a 9-month-old child (A. I.) infected with a smallpox-like disease in the village of Bokenda, Basankusu Province, the Democratic Republic of the Congo (4). Transmission from humans to animals has not been documented. While transmission from person to person is a major problem, it mostly occurs through the respiratory tract by sneezing, coughing, large respiratory droplets, and other similar actions. Direct contact with infected lesions and bodily fluids or indirect contact with contaminated items like patient-used garments or linens (17). In addition to the placenta (congenital monkeypox), intimate contact during labor and after delivery can also result in mother-to-child transmission (18). Currently, with an increasing number of transmissions of Health-Care Associated Infections (HCAI) reported, infection of healthcare workers has recently garnered attention. Prolonged exposure to patients increases the risk of infection among hospital staff and family members (2). According to the European Centre for Disease Prevention and Control, the 2021 incident in the UK was the first chain of transmission to be reported in Europe without epidemiological links with western and central Africa. It was also the first instance documented among males who had sex with men (MSM) (19). Infection preferentially occurs in gays, bisexuals, the MSM community, and other populations more susceptible to other sexually transmitted diseases, such as human immunodeficiency virus/acquired immunodeficiency syndrome (18, 20).

## 3 Pathophysiology

MPV can infect the human body through intradermal, mucosal, oropharyngeal, and nasopharyngeal routes after contact with susceptible people. After replication in the inoculated site for an appropriate 6-13 days, they spread to regional lymph nodes and then entered the circulation system (also called viremia) (21). The viruses in the blood infect host cells to spread to multiple sites and exhibit immunomodulation ability to escape immunosurveillance caused by horizontal gene transfer (22, 23). Although viral tropism in human tissue has not been well established, numerous animal models provide important clues regarding this scientific question. Osorio et al. detected MPV antigens in the lung, liver, heart, brain, kidney, ovarian, and pancreatic tissues of severely immunodeficient mice through immunohistochemical and histopathological tests (24). Moreover, the histopathological results of infected cynomolgus monkeys (*Macca fasicularis*) exhibited viral accumulation in the salivary epithelium, sebaceous and follicular tissue of the lip, especially in lymphoid tissues (25). Numerous animal reservoirs (e.g., dormice, prairie dogs, giant pouched rats) and various infected tissues reveal that MPV has a wide spectrum tropism, making it difficult to identify specific tissues as a site of infection (26).

**Figure 2:**
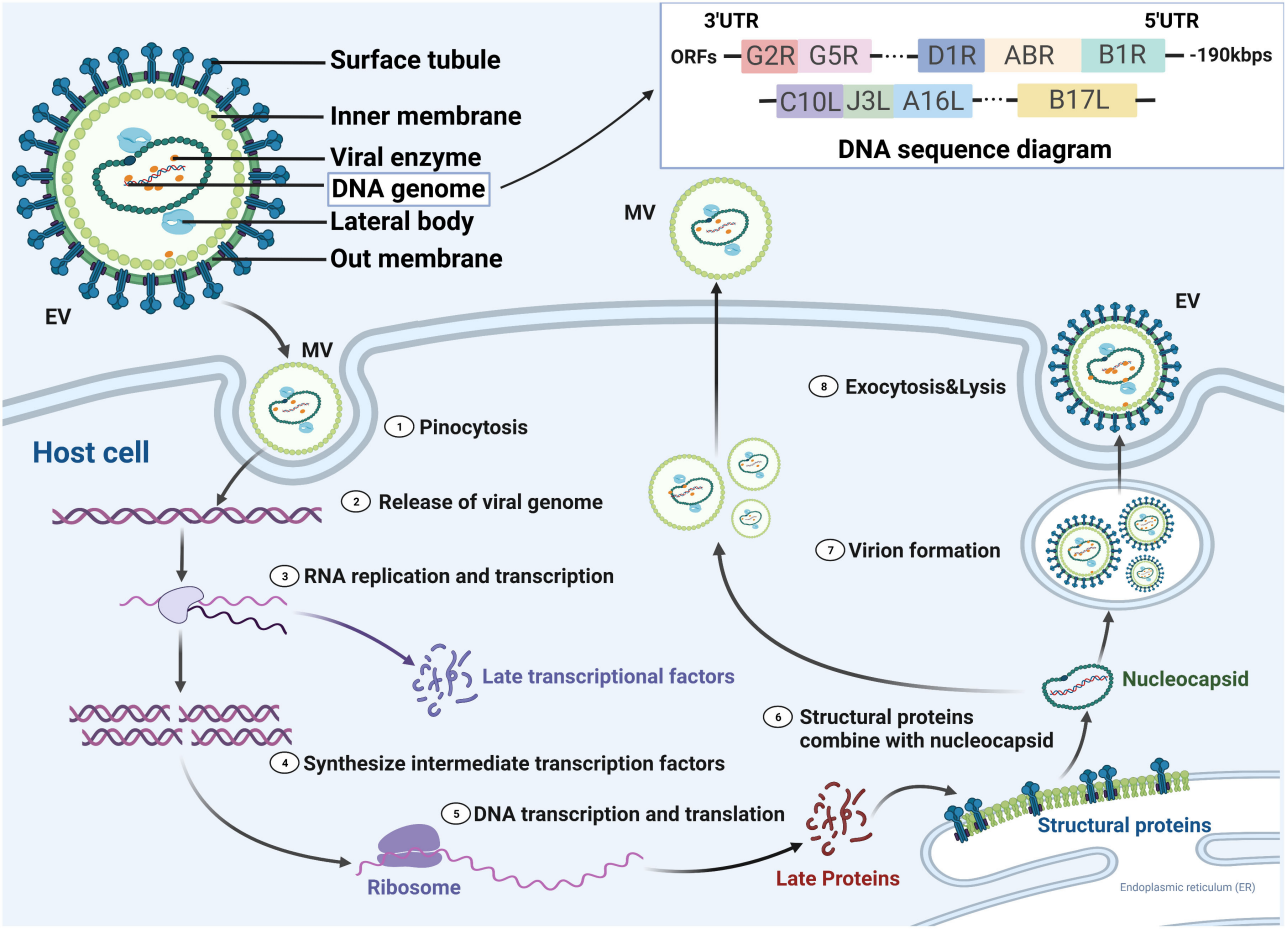
MPV life cycle in susceptible host cells. There are two forms of the virus, enveloped virion (EV) and mature virion (MV), which enter the host cell by fusion and micropinocytosis, respectively. MPV genomic structure and compositions are linear double-stranded DNA with nearly 190 kilobase pairs and contained more than 190 open reading frames (ORFs). 5′- and 3′- ends of the genome were inverted terminal repetitions, which formed hairpin-like structures. Double-stranded DNA is released and partially translated into early proteins that are processed into polymerases and immune modulators, while the others undergo replication in the cytoplasm. The resultant DNA partially transcripts into RNA, and the other part translates into intermediate proteins that are transformed into late transcription factors. Viral proteins translated from cytoplasmic RNA together with replicated DNA are assembled into nucleocapsid proteins and then processed into MVs and EVs, which are transported to the cell membrane for exocytosis. UTR, untranslated region.

MPV has structural and functional similarities to other OPVs. An exterior lipoprotein membrane with a geometric pattern of corrugations surrounds the virus’s ovoid or brick-like particle (8). The outer membrane wraps around and protects a tight core and membrane bonds that contain linear double-stranded DNA (197 kb), transcription factors, and enzymes from external stress (27). The genes essential to housekeeping activities are situated in the middle area and are highly conserved, while the genes necessary for virus-host interactions (required for virulence) are found in the terminal area and are less conserved among OPVs (28–31). Although MPV is a DNA virus, its replication, assembly, and maturation are all completed in the cytoplasm of host cells (**Figure 2**). Furthermore, most of the characteristics of the life cycle of VACV are likely to be common to MPV even though the life cycle of MPV is not well understood (30). In VACV (and probably most MPV), there are two types of infectious virions: intracellular mature virus (IMV) and extracellular-enveloped virus (EEV) generated from infected host cells (32, 33). IMVs enter the host cell through activation of macropinocytosis, while EEVs through membranous fusion (34). When compared to EEVs, which are hypothesized to facilitate viral dissemination within an infected host, IMVs are the more common infective type and mediate host-to-host transmission. For the EEVs, they continuously released from infected cells and are believed to spread locally from tissue to tissue through bodily fluids (35). Although the fact that the aforementioned characteristics are for VACV, they likely apply to all OPVs. Conversely, different agents exist among OPV species. Most OPVs like the cowpox virus evade immune function through downregulation of MHC expression of antigen-presenting cells such as monocytes. While MPV demonstrated the ability to inhibit T-cell responses against other viruses through anti-CD3 stimulation (36).

## 4 Global prevalence, from the past to present

**Figure 3:**
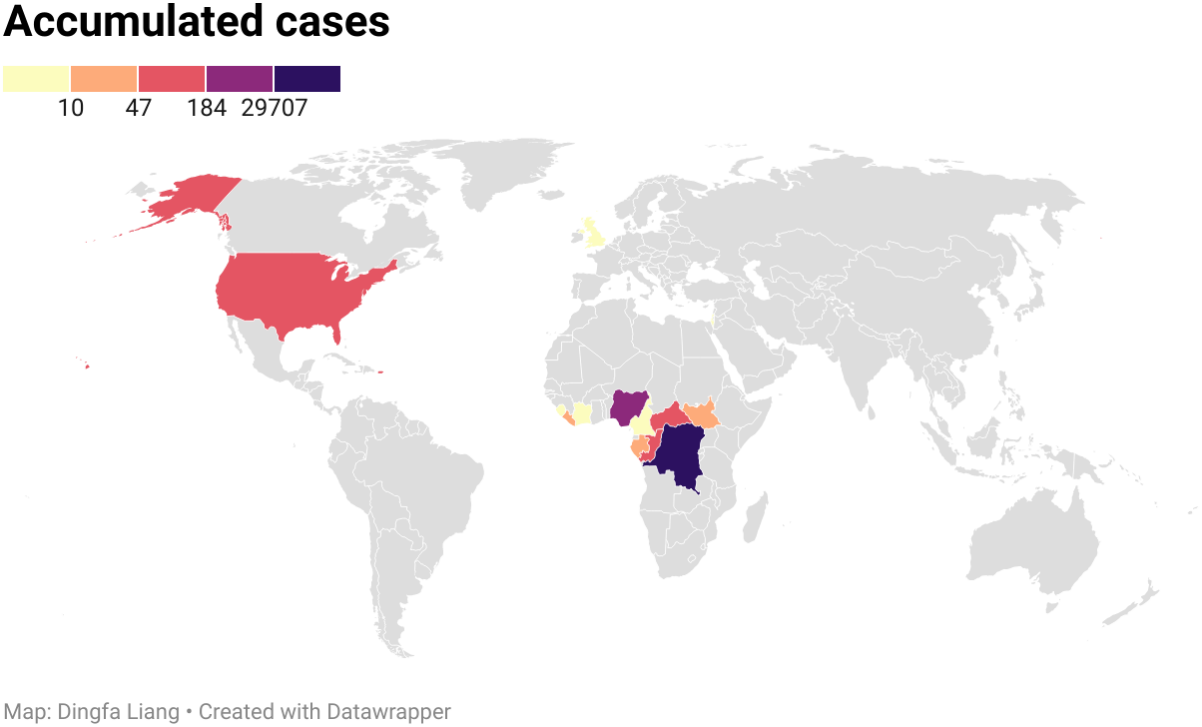
Accumulated cases of MPV from 1970 to 2020. The disease spread within 15 countries, with the Democratic Republic of the Congo being the worst hit area by the epidemic, followed by Nigeria.

Monkeypox is not a novel event and has been known as a zoonotic virus for more than 50 years. As mentioned earlier, in 1958, MPV was first found in an incidence of pox-like symptoms in monkeys at a shipment, which gives the name “monkeypox” (8). But now, it is inappropriate since MPV can infect various species and the majority of animal reservoirs are rodents, including giant pouched rats and squirrels. After the introduction of the virus by an imported animal trading company, there were at least 14 different kinds of rodents infected (37). Correspondingly, in 1970 a 9-month-old boy in the Bukenda village of the Zaire Equatorial region (now DRC) was documented to have the first human case (4). Since then, sporadic, intermittent cases of monkeypox limited in the African continent have been transmitted from local wild animals to humans. The accumulated cases are shown in **Figure 3**. From 1970 to 1979, 48 confirmed cases were detected in 6 African nations, including DRC (n=38), Cameroon (n = 1), Cote ˆ d’Ivoire (n = 1), Liberia (n = 4), Nigeria (n = 3), and Sierra Leone (n = 1) (18). In the 1980s, over 400 patients were documented, with an appropriate 10% fatality and a nine-fold increase in confirmed and probable cases recorded, and 14 infected patients were found in four additional African nations (38, 39). During the 1990s, 511 infected patients were found in DRC alone, and small outbreaks emerged across equatorial West and Central Africa (39). In 2003, 47 probable and confirmed patients of MPV infection were documented in six states of the USA who might have been exposed to prairie dogs kept as pets. The infected prairie dogs were kept in the same enclosure as Ghanaian small mammals. This was the first time that MPV occurred outside of Africa (40). From 2010 to 2019, increasing reports were documented in seven African nations (CAR, DRC, Cameroon, Nigeria, Liberia, Sierra Leone, and Republic of the Congo), the United Kingdom, Israel, and Singapore compared to past decades (39). On July 15, 2021, a patient with MPV traveling from Nigeria to the United States was found and controlled by the Texas Department of State Health Services and the CDC. More than 200 people contacted the patient and had the potential to be infected by this disease. Fortunately, there was no additional case in early September, and the contacted people were proven to be without MPV (18). However, in May 2022, a new monkeypox outbreak expanded in several countries on almost every continent. As of 14 July 2022, 1,856 laboratory-confirmed patients were documented in the UK. Among them, 12 were from Northern Ireland, 20 were from Wales, 46 were from Scotland, and 1,778 were from England (41). Given that the number of cases in the past few decades has already surpassed the whole number of infections in the first 40 years following the detection of monkeypox, it is apparent that the transmission rate of the disease is rapidly rising. The transmission between humans is increasing exponentially due to adoption by gene loss, instead of progressive mutation such as COVID-19 (42) (**Table 1**). This phenomenon is a reminder of the outbreak, indicating that prevention of monkeypox transmission and urgent treatment are essential.

**Table 1:** The differences between COVID-SARS CoV2 and Monkeypox.

	Monkeypox	COVID-19
Type of virus	Monkeypox virus (2)	SARS-CoV-2 (43)
Major transmission routes	Respiratory droplets and sexual contact (18)	Liquid droplets and contaminated contact (44)
Cell tropism	Lymphocytes, surface epithelia (45)	Respiratory epithelia and alveolar macrophages (46)
Genetic revolution	Gene loss (42)	Progressive mutation (47)
Incubation time	6 to 13 days (21)	2 to 14 days (48)
Major manifestations	Skin rash, fever, adenopathy and chills (38)	Fever, headache, nausea, cough, muscle pain, taste or smell loss, fatigue, proteinuria, hematuria, depression (43, 49)
Confirmed cases/deaths	85,536/91 (41)	753,258,129/6,811,531 (50)
Treatments	Antiviral treatment: Tecovirimat, Cidofovir, Brincidofovir (51–53) Vaccination: ACAM2000, VIGIV (54)	Antiviral treatment: Nirmatrelvir with Ritonavir, Remdesivir, Molnupiravir (55)

SARS-CoV-2: severe acute respiratory syndrome coronavirus 2;

The number of total confirmed cases and deaths was up to February 1 in 2023.

**Figure 4:**
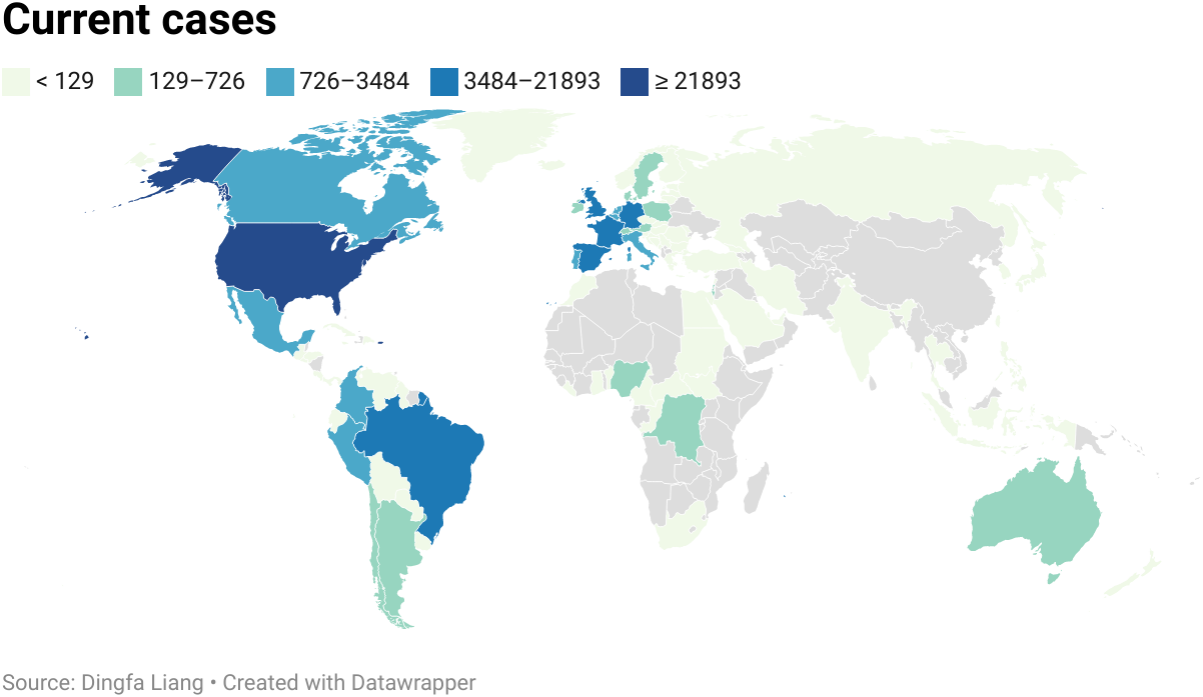
Current cases of MPVX from 1 Jan. to 9 Sep. 2022. More than 57,000 human cases spread globally in more than 100 locations. The United States of America was the worst-hit area, with 2,1893 cases.

MPV infection in humans is currently a growing public health concern as more than 85,000 clinical infections of MPV infection have been published in more than 100 different countries around the world, compared to only 48 cases in the 1970s (18). And the continuing monkeypox outbreak was regarded as a general populace crisis of international concern by the WHO on July 23, 2022 (56). As opposed to other outbreaks connected to travel from endemic nations or contact with animals transported in from the affected area, the infection source has not been identified as of yet (23). It was reported that patients with monkeypox in the current outbreak generally had close, prolonged physical contact with other monkeypox patients. During investigation, many reports documented that approximately 98% of these patients are gays, bisexuals, or other MSM (57–59). According to CDC data published on February 1, 2023, 84,243 cases were located in 103 distinct places that had never before recorded monkeypox, such as Spain (7,528 cases) and Germany (3,692 cases) (60) (**Figure 4**). A total of 1,293 human patients were documented in 7 places where monkeypox was historically reported, such as Nigeria (775 cases) and the DRC (348 cases). The total number of MPV cases reached 85,536 on February 1 in 2023, with the most of cases accumulating in Europe, the USA, and South America (41) (**Table 2**).

**Table 2:** The current cases of MPV until 1 Feb. 2023 (41).

Country	Total confirmed cases
Central African Republic	20
Ghana	121
Cameroon	18
Democratic Republic of the Congo	348
Republic of the Congo	5
Liberia	6
Nigeria	775
Aruba	3
Andorra	4
United Arab Emirates	16
Argentina	1,075
Australia	144
Austria	327
Belgium	793
Benin	3
Bulgaria	6
Bahrain	1
Bahamas	2
Bosnia and Herzegovina	9
Bermuda	1
Bolivia	264
Brazil	10,745
Barbados	1
Canada	1,460
Switzerland	551
Chile	1,416
China	1
Colombia	4,072
Costa Rica	140
Cuba	8
Curaçao	3
Cyprus	5
Czechia	71
Germany	3,692
Denmark	196
Dominican Republic	52
Ecuador	483
Egypt	3
Spain	7,528
Estonia	11
Finland	42
France	4,128
United Kingdom	3,735
Georgia	2
Gibraltar	6
Guadeloupe	1
Greece	86
Greenland	2
Guatemala	348
Guyana	2
Hong Kong	1
Honduras	13
Croatia	33
Hungary	80
Indonesia	1
India	22
Ireland	228
Iran	1
Iceland	16
Israel	262
Italy	954
Jamaica	18
Jordan	1
Japan	15
South Korea	4
Lebanon	26
Sri Lanka	2
Lithuania	5
Luxembourg	57
Latvia	6
Saint Martin	1
Morocco	3
Monaco	3
Moldova	2
Mexico	3,768
Malta	33
Montenegro	2
Mozambique	1
Martinique	7
New Caledonia	1
Netherlands	1,260
Norway	95
New Zealand	41
Panama	118
Peru	3,727
Philippines	4
Poland	215
Portugal	951
Paraguay	82
Qatar	5
Romania	47
Russia	2
Saudi Arabia	8
Sudan	18
Singapore	21
El Salvador	88
San Marino	1
Serbia	40
Slovakia	14
Slovenia	47
Sweden	260
Thailand	13
Turkey	12
China	4
Ukraine	5
Uruguay	19
United States	30,123
Venezuela	12
Vietnam	2
South Africa	5

## 5 Clinical manifestations

**Figure 5:**
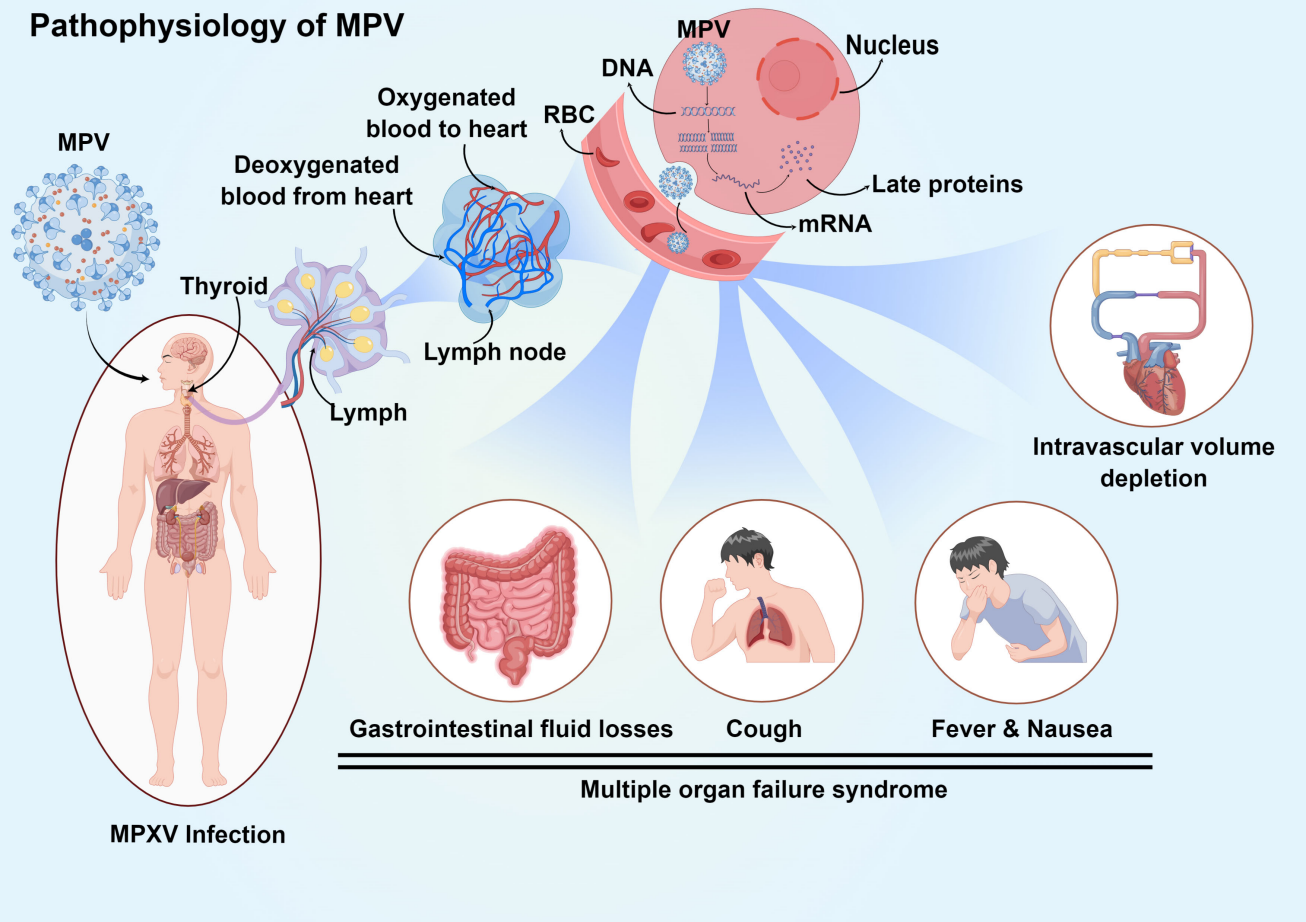
Pathophysiology and clinical manifestation of systemic MPV infection. Infection initiates in the upper respiratory tract and progresses to lymph, and then the virus enters the bloodstream through lymphocytes. The MPV in the blood spreads through the circulatory system to all parts of the body, enters the cell through endocytosis, releases DNA, and uses the substances in the cell to transcribe proteins, finally affecting the normal physiological function of the cell. MPV causes lymphocytosis and leukocytosis, thrombocytopenia, elevated aminotransferase levels, and decreased blood urea nitrogen levels. At the same time, symptoms such as multiorgan inflammation and cough and fever also occur.

From infection to the beginning of clinical manifestations, the incubation period of MPV was estimated to be 12 days and may extend to 21 days in some cases (40). The first signs or symptoms of patients include rash, fever, cough, headache, nausea and/or vomiting, confusion, wheezing, chills, sweats, runny nose, red eyes, stiff neck, lymphadenopathy, shortness of breath, sore throat, joint pain, back pain, chest pain, abdominal pain, myalgia, and conjunctivitis (38) (**Table 3**). It was reported that patients initially presented with signs or symptoms, such as rash (97%), fever (85%), adenopathy (71%), and chills (71%), which constituted the initial syndrome of MPV infection. The fever lasted for an average of 8 days (range: 2-13 days), and the rash continued for an average of 12 days (range: 7-24 days). The median time interval between the start of the fever and the appearance of the rash was 2 days (range: 0-12 days). Other signs and symptoms were evident between 0 and 14 days after the onset of fever or rash (62) (**Figure 5**).

**Table 3:** Laboratory/clinical manifestations in MPV patients.

Organ system involved	Laboratory/clinical manifestation
Blood	• lymphocytosis, leukocytosis, and thrombocytopenia (40) • hypoalbuminemia, low blood urea nitrogen level, and high transaminase levels (40) • hemorrhagic pustular lesions (61) • significant loss of intravascular volume (61)
Lymph	• swollen lymph nodes (62) • affects the oral mucosa, skin, thymus, spleen, reproductive system, and gastrointestinal tract by spread from viremia (63)
Gastrointestinal	• vomiting, diarrhea, and dehydration (54) • hypoalbuminemia and gastrointestinal fluid losses (40)
Skin mucous membranes	• rash, spots, papules, blisters and pustules (9) • areas of erythema or hyperpigmentation of the skin • ulcerative or necrotic lesions (64)
Genitals	• rash (7) • infectious sores (7) • spread by sexual contact (65)
Brain/Neurological	• encephalitis • headache (66)
Lung/Respiratory Pulmonary	• massive retropharyngeal abscess (67) • compromised tracheal airway (61)
Eyes	• keratitis and corneal ulceration (68)

### 5.1 End organ diseases

#### 5.1.1 Skin mucous membranes

The rashes with various sizes appear within 1 to 5 days following the commencement of the fever, initially on the face and then spreading to include the hands, feet, and legs (69). The rash progresses through several stages, from spots and papules to blisters (fluid-filled blisters) and pustules, before gradually going away as crusts and scabs wear off over time (38). Sixty-eight percent of patients had monomorphic lesions, and 48% of patients had centrifugally distributed lesions. Ulcerative or necrotic lesions are reported in 25% of patients, with a very small number presenting as hemorrhagic pustules (62). Different stages of the rash may appear simultaneously, and areas of skin erythema or hyperpigmentation are usually found as discrete lesions (70). Once the prodromal symptoms or rash appear, patients are considered infectious until the lesion crusts and the crusts fall off (71).

#### 5.1.2 Blood

Recent studies have shown that in addition to lymphocytopenia and thrombocytopenia observed in over one-third of evaluable patients, leukocytosis, low blood urea nitrogen levels, high transaminase levels, and hypoalbuminemia are common symptoms during the disease (72, 73). Only 2 MPV individuals had hemorrhagic pustular lesions, according to a review of 34 verified patients in the USA. Disseminated intravascular coagulation has not been reported by any individuals, and the thrombocytopenia was typically minor (40).

#### 5.1.3 Lymph

Despite the symptoms and lesions of monkeypox being difficult to distinguish from those of smallpox, the sign and symptoms of MPV infection are less severe. Up to 90% of MPV patients suffered from lymphadenopathy, which is thought to be a clinical characteristic that separates human monkeypox from smallpox (74). The appearance of swollen lymph nodes, especially in the inguinal, submental, submandibular, and cervical nodes, distinguishes monkeypox from smallpox and chickenpox (75). Meanwhile, the lymphatogenous spread of MPV caused by viremia affects the skin, spleen, thymus, oral mucosa, reproductive system, and gastrointestinal tract in monkeys that have been experimentally infected with aerosolized MPV (26).

#### 5.1.4 Gastrointestinal tract

According to case studies, MPV-infected individuals also suffer from gastrointestinal disorders, including vomiting, diarrhea, and dehydration (40). Infected individuals with mucosal and gastrointestinal clinical manifestations may require volume replenishment due to gastrointestinal losses caused by poor nutritional balance or negative protein. Gastrointestinal fluid losses and hypoalbuminemia are associated with the fluid transfer from intravascular to extravascular compartments that takes place in systemic illness (76).

#### 5.1.5 Genitals

A rash normally begins on the face but can extend to other tissues, including the genitalia (19). According to the European Centre for Disease Prevention and Control, many cases in the ongoing outbreak are associated with sexual contact, particularly with men who were identified as gay, bisexual, or males who have sex with other men (58). Furthermore, the transmission of viruses can be achieved *via* sharing clothing and bedding as well as being directly exposed to infectious ulcers, scar tissue, or bodily fluids (19). Although less severe, the symptoms of monkeypox are similar to those of smallpox and consist of a characteristic rash that is preceded by mild prodromal manifestations (including lymphadenopathy, fever, and flu-like symptoms) (7). The cases in the present outbreak are characterized by a distinct rash that begins in the vaginal and perianal regions and extends to other parts of the human body with or without it (40).

#### 5.1.6 Other clinical complications

A series of complications reported that 5 cases were identified as severely ill, and 9 cases were hospitalized as inpatients in the United States. Among those cases hospitalized as inpatients, a 6 years old child with encephalitis required intubation and mechanical breathing, while a 10-year-old girl with severe cervical lymphadenopathy and a retropharyngeal abscess had a constructed tracheal airway (77, 78). The intensive care units were allowed for these two patients. In addition, there was a patient with comorbidity (hepatitis C) who had a serious illness and was kept in the hospital as an inpatient. The individual is fully rehabilitated without experiencing any negative consequences. One adult with infection-related issues was found to have bacterial superinfection (unknown microorganism) (79), while another had keratitis and corneal erosion, which eventually necessitated a corneal transplant (40). There was no underlying medical condition in any of these patients. These cases show that monkeypox can cause a range of complications in addition to direct disease. This further illustrates the dangers of monkeypox and the need for prevention and control and provides another way of thinking about reducing the dangers of monkeypox, namely, the prevention of complications.

### 5.2 Diagnosis

Tests for diagnosing diseases are of vital importance for the confirmation of MPV infection. Medical history, clinical symptoms, and laboratory tests all contribute to the diagnosis of MPV infection. The enlargement of the lymph nodes is the most typical symptom that separates monkeypox from diseases such as smallpox and chickenpox, which also present with rash symptoms, while confirmation of monkeypox also requires laboratory test assistance (80). Swabs are used to collect crusts or exudates from the infective site to isolate viral nucleic acids for diagnostic purposes. This is followed by an MPV genome-specific real-time polymerase chain reaction (RT-PCR) assay to detect viral DNA (71). Moreover, western blot (WB) analysis using MPV proteins can also be used to confirm MPV infection. WB has lower requirements for detection equipment or laboratories and the detection results are more accurate, but there are some limitations compared with RT-PCR. Such as infection cannot be detected until 7 days after the infection; Blood collection is required and the operation is relatively difficult; not suitable for application to the large-scale collection, etc. (81, 82). So according to the WHO, the preferred test for identifying MPV during acute infection is the RT-PCR test (83). If the mother is infected by MPV, the fetus requires ultrasound surveillance to determine if he/she is infected with MPV by examining the existence of ultrasound anomalies such as fetal hepatomegaly or hydrops (84).

## 6 Treatments

Most people recover from the disease without treatment and the symptoms of monkeypox are typically mild (54). Despite the lack of specific therapies for monkeypox, studies have shown that the smallpox vaccine has an 85% success rate in preventing monkeypox. Furthermore, several antiviral drugs may also be effective in treating monkeypox infections (63) (**Table 4**).

**Table 4:** A list of treatments for monkeypox management.

Treatments	Route	Dosing	Biologic and/or clinical efficacy	Adverse events	Contraindications	Use in specific populations
Vaccinia Immune Globulin Intravenous	IV	Adults:6000 U/kg (9000 U/kg might be taken into consideration if the patient doesn’t respond to the first dose); Pediatrics: Children should not use this medication because it has not been proven safe and effective in people under the age of 18 (7).	Passive immunity is provided by antibodies derived from the combined human plasma of people who have received the smallpox vaccination (85)	Dizziness, rigors, nausea, headache (86)	An IgA deficit with antibodies against IgA, a history of IgA hypersensitivity, a history of severe systemic or anaphylactic reactions to human globulins, and isolated vaccinia keratitis (7).	Use caution in pregnant women and patients with renal insufficiency (52, 86).
Tecovirimat	PO, IV	For adults, take 600 mg twice daily for 14 days. For children (13 kg to less than 25 kg), take 200 mg BID for 14 days. For those between 25 and 40 kg, take 400 mg BID for 14 days. For those over 40 kg, take 800 mg BID for 14 days. 14 days at 600 mg twice a day (87).	Restrain orthopoxvirus VP37 envelope wrapping protein (88).	Headache, nausea, abdominal pain, vomiting (89).	Have not reported.	PO: Hepatic/renal adjustment not needed. IV: should not be administered to patients with severe renal impairment (87).
Cidofovir	IV	5 mg/kg once weekly for two weeks, followed by 5 mg/kg IV once every other week (90).	Selectively inhibits orthopoxvirus DNA polymerase-mediated viral DNA synthesis by cellular phosphorylation (53).	Diarrhea, nausea, vomiting, and abdominal pain (86).	Serum creatinine > 1.5 mg/dL; urine protein ≥ 100 mg/dL (≥ 2+ proteinuria); history of clinically severe hypersensitivity to probenecid or other sulfa-containing drugs; CrCl ≤ 55 mL/minute (87).	It is required to change the dosage based on renal function (91).
Brincidofovir	PO	Adults over 48 kg should take 200 mg once per week in two doses. Adults and pediatric patients between 10 kg and 48 kg should take 4 mg/kg of oral suspension once per week in two doses. Pediatric patients under 10 kg should take 6 mg/kg of oral suspension once per week in two doses (92).	Cidofovir diphosphate selectively inhibits orthopoxvirus DNA polymerase-mediated viral DNA synthesis (93).	Fever, infection, proteinuria, iritis, hypotony of the eye, decreased serum bicarbonate, uveitis, neutropenia, nephrotoxicity (87)	Have not reported.	Not recommended in pregnant and breast-feeding women.

BID twice a day, IV intravenous, PO per os (orally), CrCl creatinine clearance, and DNA deoxyribonucleic acid.

### 6.1 Smallpox vaccine (ACAM2000)

The US CDC has approved the use of ACAM2000 in emergencies, and it can offer 85% cross-protective resistance against monkeypox (54). Patients must be informed of the abnormal risk of fetal vaccinia by ACAM2000 if high-risk monkeypox exposure occurs during pregnancy. Fetal vaccinia can cause stillbirth, neonatal death, premature delivery, and possibly unfavorable maternal reactions (94). The third-generation MVA-BN (Modified Vaccinia Ankara-Bavarian Nordic) smallpox vaccine, which has received universal approval within the EU, Canada, and the USA, may be gentler because it comprises the virus that cannot replicate and has not been proven to cause problems during pregnancy (95).

### 6.2 Tecovirimat (TPOXX/ST-246)

Tecovirimat (TPOXX/ST-246), an FDA-approved antiviral medicine, is used to alleviate both pediatric and adult patients with human smallpox illnesses. It acts as a suppressor of the OPV VP37 envelope protein and can be administered as a pill or injection (51). The capsule can be opened and the drug mixed with semisolid food for kids under the weight of 12.9 kilograms (96). Although studies on numerous animal species have shown that tecovirimat is beneficial in treating diseases brought on by OPVs, it is still unclear if tecovirimat is effective in treating human monkeypox infections. One case study was an adult patient who received tecovirimat and made a full recovery after spending a month in the hospital (97), while in another case study, a patient underwent oral tecovirimat (200 mg twice a day for two weeks) treatment without experiencing any negative side effects (67). The CDC has an enhanced access protocol, also known as a “compassionate use” approach, that allows the application of tecovirimat for the mitigation of monkeypox when an outbreak occurs (98, 99).

### 6.3 Vaccinia immune globulin intravenous

The FDA has authorized the use of VIGIV, a hyperimmune globulin, to treat the side effects of vaccinia-related conditions such as progressive vaccinia, severe generalized vaccinia, *dermatitis vaccinatum*, vaccinia infections in persons with skin issues, and abnormal infections brought on by VACV (except in cases of isolated keratitis). The application of VIGIV for the therapy of OPVs outbreak such as monkeypox is permitted by the CDC’s expanded access protocol (100). Although it is a potential strategy, there is little data on how VIGIV acts against smallpox and monkeypox, and the utilization of VIGIV for smallpox or monkeypox has not been tested in humans. However, in extreme circumstances, medical professionals might consider their use incurring monkeypox. Notably, individuals with severely compromised T-cell functionality should not be vaccinated against the MPV. Instead, VIGIV may be administered to them if they have a history of exposure (101).

### 6.4 Cidofovir (Vistide)

The antiviral medicine Cidofovir (Vistide), which blocks viral DNA polymerase from functioning, has been proven to be effective against poxviruses *in vitro* and in preclinical studies (52). It is an antiviral medication that the FDA has approved for use in treating cytomegalovirus (CMV) retinitis in AIDS patients. The effectiveness of Cidofovir in treating monkeypox in people is not well understood. However, investigations on animals and *in vitro* have shown that it is effective against OPVs (102). To mitigate OPV (including monkeypox) in an outbreak, the CDC has an enhanced access protocol that permits the utilization of stocked cidofovir. Cidofovir treatment may be implemented in cases with severe monkeypox infection, although it is unknown whether such a patient will benefit from it. Cidofovir may be less safe than Brincidofovir since Cidofovir can cause serious renal damage or other side effects (90). Currently, the CDC is working on an Expanded Access Investigational New Drugs to enable the drugs to be used for the treatment of monkeypox. They could pick the CMX-001 medication, a modified version of cidofovir, which has demonstrated antiviral activity against OPV species but lacks the level of nephrotoxicity typically associated with cidofovir (103).

### 6.5 Brincidofovir (Tembexa)

On June 4, 2021, FDA licensed Brincidofovir (Tembexa) as an antiviral drug to alleviate human smallpox in newborns, children, and adults. Although the small numbers involved make it difficult to extrapolate the effectiveness of Brincidofovir in the therapy of monkeypox infection, their efficacy against OPV has been illustrated *in vitro* and animal models (53, 104). However, a review of patients enrolled since the launch of the HCID (airborne) network between August 15, 2018, and September 10, 2021, revealed that all 3 adult patients receiving oral Brincidofovir (200 mg once a week) had high hepatic enzymes, which led to the termination of therapy (67). The CDC is now working on an EA-IND (Expanded Access Investigational New Drug) to make it easier to utilize Brincidofovir as a monkeypox treatment.

## 7 Conclusion and future perspectives

Monkeypox is no longer “a viral zoonotic disease that occurs mainly in remote portions of Central and West Africa, near tropical rainforests”, as the expansion of the disease over the past several years and the ongoing outbreak (105). Although most MPV infections cause self-limited and mild diseases, with supportive treatment being relatively sufficient, there is no specific therapy available for it to date, and it can be transmitted in a variety of ways, such as droplets, contact, and sexual contact. Its possibility for additional regional and worldwide spread is therefore still a serious concern. The majority of information on the illness is gathered from individual cases or outbreak reports, as well as from passive sporadic monitoring, none of which provides a complete overview. To effectively lead data collection, prevention, preparedness, response and surveillance efforts for monkeypox and other emerging or re-emerging diseases with pandemic possibility, there is still an urgent need to improve surveillance and public health skills.

Numerous antiviral drugs that may be beneficial in the mitigation of monkeypox were authorized for the treatment of smallpox based on model studies, such as Tecovirimat, VIGIV, Cidofovir, and Brincidofovir, but the efficacy of these agents has not been completely characterized; thus, more research on these therapies in humans is needed. In addition, there are several variables can affect the prognosis of monkeypox, such as prior vaccination records, baseline health state, and co-occurring illnesses or comorbidities. Therefore, the most sensible course of action is to design treatments specifically for each patient in accordance with their likelihood of contracting a severe illness.

## Author contributions

Conceptualization and design: XL and PY; data acquisition and writing-original draft preparation, LN and DL; data analysis and interpretation: JZ and ZL; revision for intellectual content: all authors. All authors agree to be accountable for all aspects of the work. All authors contributed to the article and approved the submitted version.
